# Age- and Sex-Dependent Modulation of Exogenous Ketone Supplement-Evoked Effects on Blood Glucose and Ketone Body Levels in Wistar Albino Glaxo Rijswijk Rats

**DOI:** 10.3389/fnins.2020.618422

**Published:** 2021-01-11

**Authors:** Zsolt Kovács, Brigitta Brunner, Dominic P. D’Agostino, Csilla Ari

**Affiliations:** ^1^Savaria Department of Biology, Savaria University Centre, ELTE Eötvös Loránd University, Szombathely, Hungary; ^2^Institute of Biology, Faculty of Sciences, University of Pécs, Pécs, Hungary; ^3^Laboratory of Metabolic Medicine, Department of Molecular Pharmacology and Physiology, Morsani College of Medicine, University of South Florida, Tampa, FL, United States; ^4^Ketone Technologies LLC, Tampa, FL, United States; ^5^Institute for Human and Machine Cognition, Ocala, FL, United States; ^6^Behavioral Neuroscience Research Laboratory, Department of Psychology, University of South Florida, Tampa, FL, United States

**Keywords:** ketone supplementation, ketone ester, β-hydroxybutyrate, aging, sex, glycemia, hyperketonemia

## Abstract

In certain disease states, such as epilepsy, the elevation of blood ketone levels with ketogenic diets (KDs) has beneficial effects, while exogenous ketone supplements (EKSs) were shown to increase the level of blood ketone bodies (such as β-hydroxybutyrate, βHB) and decrease blood glucose levels without dietary restrictions. It has been suggested that ketone body and glucose utilization of the body may be modified by age and gender resulting in changes in blood βHB and glucose levels, but it was not investigated through several months yet. Thus, we investigated whether the effect of an EKS on blood βHB and glucose level is modulated by age and sex in Wistar Albino Glaxo Rijswijk (WAG/Rij) rats, a model animal of human absence epilepsy. We used KEMCT (1:1 mix of ketone ester/KE and medium-chain triglyceride/MCT oil) by oral gavage in female and male WAG/Rij rats. Animals were fed with standard diet, which was supplemented by KEMCT (2.5 g/kg) once per month by oral gavage for 17 months. One hour after KEMCT treatment, changes in blood R-beta-hydroxybutyrate (R-βHB) and glucose levels were measured. KEMCT gavage significantly increased blood R-βHB and decreased blood glucose levels, in both male and female rats, compared with the controls. In male rats, the KEMCT-induced increase in blood R-βHB levels was lower at the 7th and 8th months and higher at the 16th and 17th months, compared with the results at the 1st month. KEMCT-generated increase in R-βHB levels was higher in female rats, compared with male rats between the 2nd and 11th months, but older (between the 14th and 17th months) female rats showed lower levels than males. KEMCT gavage induced significantly lower glucose levels at the 4th, 9th, 10th, 12th, and 13th months in both sexes, but between the 14th and 17th months, only males showed significantly lower levels, compared with the results at the 1st month. KEMCT treatment induced lower blood glucose levels in female than in male rats between the 1st and 8th months, but higher glucose levels were measured in female rats at the 17th month than in males. These findings suggest that age and sex can modify the EKS-evoked effects on blood R-βHB and glucose concentrations.

## Introduction

Emerging evidence suggests that ketogenic diet (KD)- and exogenous ketone (ketogenic) supplement (EKS)-evoked ketosis may have a modulatory role in physiological and pathophysiological processes of the central nervous system (CNS). For example, ketone bodies, such as β-hydroxybutyrate (βHB) and acetoacetate (AcAc), provide fuel to brain cells for mitochondrial ATP synthesis, modulate functioning of neurotransmitter systems (e.g., glutamatergic, GABAergic, and adenosinergic systems) and ion channels (Newman and Verdin, 2014; Sharma et al., 2015; Achanta and Rae, 2017), and enhance (generate) neuroprotective effects (e.g., decrease the production and release of reactive oxygen species and pro-inflammatory cytokines, such as interleukin-1β) (Maalouf et al., 2009; Youm et al., 2015; Norwitz et al., 2019). Consequently, it has been demonstrated that ketone bodies may have therapeutic potential in the treatment of several CNS diseases, such as epilepsy, neurodegenerative diseases (e.g., Alzheimer’s disease and Parkinson’s disease), and psychiatric disorders (e.g., anxiety and depression), likely through ketosis-evoked neuroprotective effects (Hashim and VanItallie, 2014; Ari et al., 2016; Kovács et al., 2019a), and other age-associated diseases (e.g., cardiovascular diseases and cancer) (Han et al., 2020).

It has also been demonstrated that not only KDs (containing high-fat, adequate protein, and restricted carbohydrate) but also EKSs, such as ketone ester (KE), ketone salt (KS), medium-chain triglyceride (MCT) oil, and their mixes (e.g., KEMCT), can evoke and maintain ketosis in both animals and humans (Ari et al., 2016; Kesl et al., 2016; Stubbs et al., 2017; Kovács et al., 2019b). Moreover, EKSs are well-tolerated and can generate therapeutic ketosis (ketone levels = 1–7 mM) while maintaining a normal diet (Clarke et al., 2012; Hashim and VanItallie, 2014; Ari et al., 2016; Stubbs et al., 2017). Consequently, EKS administration-generated therapeutic ketosis may be a safe alternative method (D’Agostino et al., 2013; Ari et al., 2016; Stubbs et al., 2017) to circumvent dietary restrictions and adverse effects by KDs (e.g., nephrolithiasis, growth retardation, constipation, and hyperlipidemia) (Branco et al., 2016) and to treat not only several CNS diseases, such as epilepsy, psychiatric diseases (e.g., anxiety), neurodegenerative disorders (e.g., Alzheimer’s disease), and cancer (Newport et al., 2015; Kovács et al., 2017, 2019a; Berk et al., 2020), but also, among others, non-alcoholic fatty liver disease, obesity, cardiovascular disease, glucose intolerance, and type 2 diabetes (Han et al., 2020).

After ingestion of EKSs, ketone bodies (e.g., βHB) can enter into several organs, such as the heart and brain, and exert their neuroprotective effects through enhancement of energy metabolism, modulation of signaling systems, activation of hydroxycarboxylic acid receptor 2, and inhibition of histone deacetylases (Sato et al., 1995; Maalouf et al., 2009; Achanta and Rae, 2017; Norwitz et al., 2019). It has been demonstrated that metabolism and utilization of ketone bodies on CNS processes may be regionally different (Hawkins and Biebuyck, 1979; Hawkins et al., 1986; Bilger and Nehlig, 1992). Moreover, KD- and EKS-generated changes in blood level of ketone bodies and glucose and, as a consequence, their effects on physiological and pathophysiological processes of different organs may be modulated by gender and age (Page et al., 1971; Buc et al., 1986; Nehlig and Pereira de Vasconcelos, 1993; Vannucci, 1994; Cao et al., 2017; Ari et al., 2019, 2020). These results suggest that the alleviating effects (e.g., neuroprotective influences) of KD- and EKS-evoked increase in ketone body levels may be age- and gender-dependent in the treatment of different diseases, such as ischemia, traumatic brain injury, and epilepsy (Freeman et al., 1998a; Rho et al., 1999; Appelberg et al., 2009). Indeed, for example, KD improved motor and cognitive recovery after traumatic brain injury only in 35-day-old rats, but not in 75-day-old rats (Appelberg et al., 2009), and the antiepileptic effect of KD was better in younger animals and children, which may be in correlation with the more efficient extraction and utilization of ketone bodies from the blood, compared with adults (Freeman et al., 1998b; Bough et al., 1999; Rho et al., 1999). These results lead to the crucial question of whether age and sex can modulate EKS-evoked changes in not only blood βHB but also glucose levels.

It has been demonstrated previously that EKS-evoked ketosis can generate beneficial (modulatory) effects on absence epilepsy (Kovács et al., 2017, 2019b), anxiety (Ari et al., 2016; Kovács et al., 2018), sleep-like effects (Ari et al., 2018; Kovács et al., 2020), and motor performance (Ari et al., 2020) in a well-investigated model animal of human absence epilepsy Wistar Albino Glaxo Rijswijk (WAG/Rij) rats (Coenen and Van Luijtelaar, 2003). Theoretically, these beneficial influences of EKSs, which are likely generated through ketosis (Ari et al., 2016, 2018, 2020; Kovács et al., 2017), can be modulated by age and gender, but systematic investigation of the putative influences of age and gender on EKS-evoked effects through several months was not carried out previously. Therefore, this study extends our previous results on EKS-evoked effects in WAG/Rij rats in order to obtain further understanding of how age and sex regulate EKS-generated changes in blood βHB and glucose levels. We investigated the alteration of EKS-evoked changes in these parameters in WAG/Rij rats of both sexes between 1 and 17 months of age, using a previously established effective dose of a ketone supplement mix (KE and MCT oil in a 1:1 ratio: KEMCT; 2.5 g/kg, gavage) (Ari et al., 2020; Kovács et al., 2020).

## Materials and Methods

### Animals

Animal treatments were carried out according to the Hungarian Act of Animal Care and Experimentation (1998, XXVIII, section 243), European Communities Council Directive 24 November 1986 (86/609/EEC) and EU Directive 2010/63/EU to use and treat animals in experimental laboratories. Experiments were approved by the Animal Care and Experimentation Committee of the Eötvös Loránd University (Savaria University Centre) and the National Scientific Ethical Committee on Animal Experimentation (Hungary) under license number VA/ÉBNTF02/85-8/2016.

Male (*n* = 8) and female (*n* = 8) WAG/Rij rats (breeding colony of WAG/Rij rats at Eötvös Loránd University, Savaria University Centre, Szombathely, Hungary) were kept in groups of three to four after weaning (on the 25th day) under standard laboratory conditions (12:12 h light–dark cycle, lights on from 08:00 a.m. to 08:00 p.m.; free access to standard rodent chow diet and water; air-conditioned room at 22 ± 2°C). All efforts were made to minimize pain and suffering and to reduce the number of animals used.

### Experimental Design

Ketone ester (1,3-butanediol acetoacetate diester) was developed by D’Agostino et al. (2013) (University of South Florida/USF, United States) in collaboration with Savind, Inc. (Urbana, IL, United States), whereas MCT oil (pharmaceutical grade; approximately 60% caprylic triglyceride and 40% capric triglyceride) was purchased from Now Foods (Bloomingdale, IL, United States).

We demonstrated previously that intragastric gavage of not only KE and MCT oil but also KEMCT (mix of KE and MCT oil in a 1:1 ratio; *ad libitum* access to normal rat chow + 2.5g/kg body weight KEMCT) effectively induced and maintained ketosis without causing side effects (Ari et al., 2016; Kesl et al., 2016; Kovács et al., 2020). Therefore, in this study, 2.5 g/kg dosage of KEMCT was administered once a month by intragastric gavage for 17 months. To familiarize the animals to the gavage method, all KEMCT gavage treatments were preceded by water gavage (2.5 g/kg) for 4 days (adaptation period). Levels of blood βHB and glucose were measured 1 h after gavage (between gavage and βHB/glucose measurements, the animals had free access to food and water). Control βHB and glucose levels were measured on the last (4th) day of the adaptation period. KEMCT supplementation (2.5 g/kg, gavage), as well as measurements of KEMCT-induced changes in blood βHB and glucose levels, was performed on the 5th day. Thus, because of the procedure of gavage, the experiments were started on 1-month-old rats. A similar protocol was repeated once every month for 17 months.

Each rat was euthanized with isoflurane after the last data collection.

### Measurement of Blood βHB and Glucose Levels as Well as Body Weight

Similar to our previous studies (Kovács et al., 2018, 2020), blood was taken from the tail vein of rats about 60 min after the gavage, and βHB and glucose levels were measured by a commercially available glucose and ketone monitoring system (Precision Xtra^TM^, Abbott Laboratories, Abbott Park, IL, United States). This instrument only measures blood levels of R-βHB. Thus, total blood ketone levels (R-βHB + S-βHB + AcAc + acetone) would be higher than we measured. Body weights of rats were also measured every month 1 day before the adaptation period.

### Statistics

All data were presented as the mean ± standard error of the mean (SEM). We compared the control level of glucose and R-beta-hydroxybutyrate (R-βHB) levels (measured on the 4th day of the adaptation period of every month) to levels, which were measured after KEMCT administration in males and females on the next (5th) day for 17 months. Glucose and R-βHB levels of the 2nd and 17th months were also compared with the levels of the 1st month. Moreover, we also compared blood concentration of glucose and R-βHB of males and females measured on every control day (4th adaptation day) and every KEMCT gavage day for 17 months. Changes in body weight by age and sex were also investigated. Data analysis was performed using GraphPad Prism version 6.0a using a two-way ANOVA with Tukey’s multiple comparisons test and Sidak’s multiple comparisons test (Ari et al., 2018; Kovács et al., 2018). Pearson correlation was calculated for KEMCT treatment-induced changes in blood βHB and glucose levels. Results were considered significant when *p* < 0.05.

## Results

### Effect of KEMCT Treatment on Blood R-βHB and Glucose Levels

**FIGURE 1:**
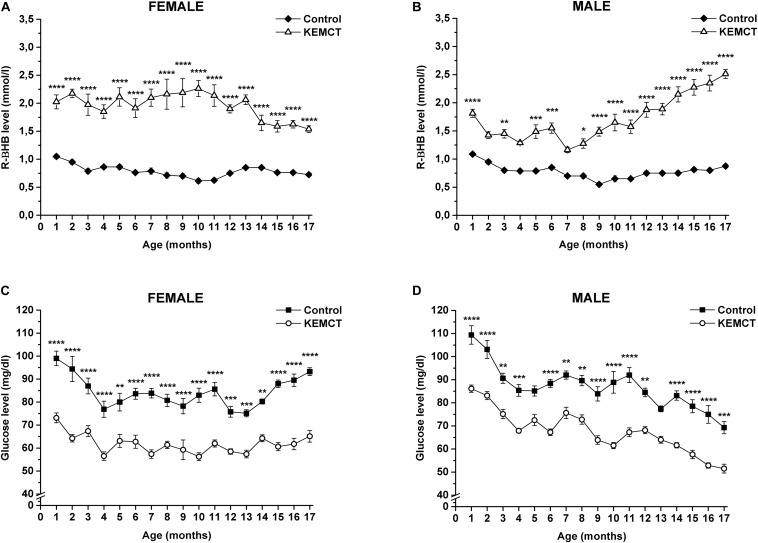
KEMCT-evoked changes in blood R-βHB and glucose levels in female and male WAG/Rij rats. KEMCT administration (gavage, 2.5 g/kg, once per month) significantly elevated the blood R-βHB levels **(A,B)** and decreased blood glucose levels **(C,D)** compared with the control in both female and male rats. R-βHB, R-beta-hydroxybutyrate; KEMCT, mix of ketone ester (KE) and medium-chain triglyceride (MCT) oil in a 1:1 ratio. *p < 0.05; **p < 0.01; ***p < 0.001; ****p < 0.0001.

Mix of KE and MCT gavage significantly increased blood R-βHB levels in both female (Figure 1A) and male (Figure 1B) WAG/Rij rats every month, compared with the control, except on the 2nd, 4th, and 7th months in male rats (however, there was a trend of increased R-βHB levels, compared with the control).

A significant decrease in blood glucose levels was demonstrated after KEMCT gavage, compared with the control every month in both sexes, except at the 5th and 13th months of male rats (Figures 1C,D).

Based on Pearson correlation analysis, blood R-βHB levels (Figures 1A,B) negatively correlated with blood glucose levels (Figures 1C,D) after KEMCT treatment in all rats (*R*^2^ = −0.71042), and separately in female (*R*^2^ = −0.73431) and male (*R*^2^ = −0.68207) rats as well, suggesting ketosis-induced hypoglycemia. A negative but weak correlation was demonstrated between control glucose levels and blood R-βHB levels after KEMCT treatment in male rats (*R*^2^ = −0.31633), suggesting that the decrease in control glucose levels (Figure 1D) may be in association with the increase in R-βHB levels after KEMCT treatment (Figure 1B) between the 11th and 17th months (*R*^2^ = −0.24827) in male rats.

### Age- and Sex-Dependent Alterations in Blood R-βHB Levels

**FIGURE 2:**
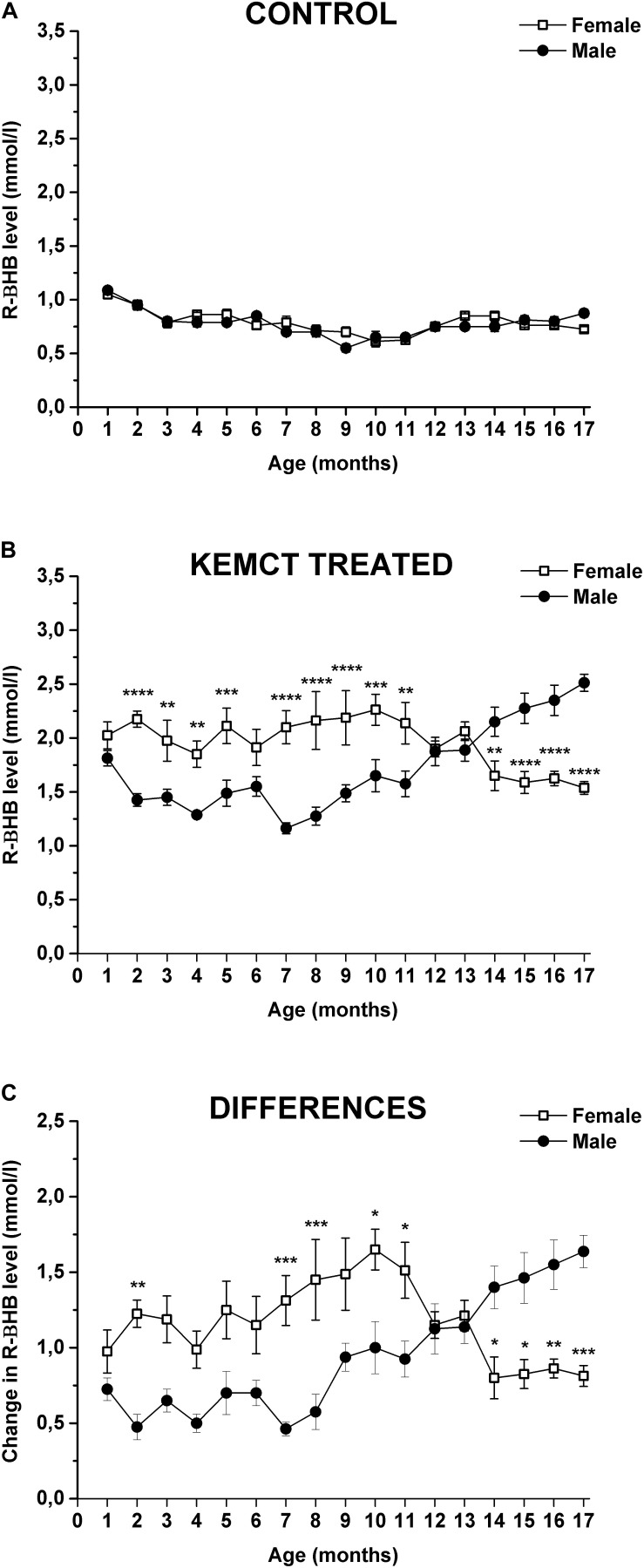
Sex-dependent alterations in blood R-βHB levels. The control level of blood R-βHB levels was similar in both sexes **(A)**. KEMCT-induced increase in R-βHB levels **(B)** and differences between KEMCT-evoked R-βHB levels and control R-βHB levels **(C)** were significantly different in female rats compared with male rats. KEMCT, mix of ketone ester (KE) and medium-chain triglyceride (MCT) oil in a 1:1 ratio; R-βHB, R-beta-hydroxybutyrate. *p < 0.05; **p < 0.01; ***p < 0.001; ****p < 0.0001.

Age and sex significantly influenced blood R-βHB levels (interaction of age and sex: *F*_33_,_476_ = 10.46, *p* < 0.0001; age: *F*_33_,_476_ = 64.90, *p* < 0.0001; sex: *F*_1_,_476_ = 28.24, *p* < 0.0001). Control blood R-βHB levels did not change significantly throughout the experiment (from the 2nd to 17th months), compared with blood R-βHB levels at baseline (1st month) in either male or female rats, except it was lower at the 9th month, than baseline in male rats (Table 1, first and second columns). Moreover, significant differences between control R-βHB levels of female vs. male rats were not identified (Figure 2A and Table 1, third column). After KEMCT gavage, similar blood R-βHB levels were demonstrated between the 2nd and 17th months, compared with baseline blood R-βHB level (1st month) in female animals (Table 1, fourth column). However, KEMCT-induced blood R-βHB levels were lower at the 7th and 8th months, whereas R-βHB levels were higher at the 16th and 17th months, compared with blood R-βHB level at baseline (1st month) in male rats (Table 1, fifth column). KEMCT gavage had different effects on R-βHB levels in female and male rats over time. KEMCT-induced increase in R-βHB levels was significantly higher in female rats than in male rats between the 2nd and 11th months (except on the 6th month), but lower R-βHB levels were measured in female rats, compared with male rats between the 14th and 17th months (Figure 2B and Table 1, sixth column). Moreover, similar results (age- and sex-dependent differences) were demonstrated when comparing differences between KEMCT-evoked R-βHB levels and control R-βHB levels (interaction of age and sex: *F*_16_,_238_ = 8.615, *p* < 0.0001; age: *F*_16_,_238_ = 3.018, *p* = 0.0001; sex: *F*_1_,_238_ = 23.33, *p* < 0.0001). These differences were higher between the 2nd and 13th months, but lower between the 14th and 17th months in female rats, compared with the baseline (1st month). In spite of that, this trend of increase and (later) decrease was demonstrated in females; however, the results were non-significant (Table 1, seventh column). A trend of decrease (between the 2nd and 8th months) and later increase (between the 9th and 17th months) in differences was demonstrated in male rats, compared with the difference at baseline (1st month) (Table 1, eighth column). These differences were significant between the 15th and 17th months. Differences between KEMCT-evoked R-βHB levels and control R-βHB levels were significantly higher in female rats than in male rats at the 2nd, 7th, 8th, 10th, and 11th months, but significantly lower differences were observed in female rats between the 14th and 17th months, compared with males (Figure 2C and Table 1, ninth column). Thus, KEMCT gavage-generated increase in blood R-βHB levels was increasingly efficient by age only in male rats (Figures 2B,C and Table 1).

**TABLE 1:** Effect of age and sex on KEMCT-evoked changes in blood R-βHB levels.

**BLOOD R-βHB LEVELS (mmol/l; mean ± SEM)**									
**Age (month)**	**Control (2.5 g/kg water, gavage)**			**KEMCT treated (2.5 g/kg, gavage)**			**Differences (KEMCT treated – control)**		
	
	**Female (significance: compared to 1st month)**	**Male (significance: compared to 1st month)**	**Female vs. male: significance**	**Female (significance: compared to 1st month)**	**Male (significance: compared to 1st month)**	**Female vs. male: significance**	**Female (significance: compared to 1st month)**	**Male (significance: compared to 1st month)**	**Female vs. male: significance**
1st	1.05 ± 0.04 (−)	1.09 ± 0.03 (−)	ns/>0.9999	2.03 ± 0.13 (−)	1.81 ± 0.07 (−)	ns/0.9864	0.98 ± 0.14 (−)	0.73 ± 0.08 (−)	ns/0.9782
2nd	0.95 ± 0.05 (ns/>0.9999)	0.95 ± 0.05 (ns/>0.9999)	ns/>0.9999	2.18 ± 0.08 (ns/>0.9999)	1.43 ± 0.06 (ns/0.9227)	****/ < 0.0001	1.23 ± 0.09 (ns/0.9975)	0.48 ± 0.08 (ns/0.9975)	**/0.0027
3rd	0.79 ± 0.05 (ns/>0.9999)	0.80 ± 0.04 (ns/>0.9999)	ns/>0.9999	1.98 ± 0.19 (ns/>0.9999)	1.45 ± 0.08 (ns/0.9885)	**/0.0043	1.19 ± 0.16 (ns/0.9996)	0.65 ± 0.08 (ns/>0.9999)	ns/0.1026
4th	0.86 ± 0.02 (ns/>0.9999)	0.79 ± 0.04 (ns/>0.9999)	ns/>0.9999	1.85 ± 0.12 (ns/>0.9999)	1.29 ± 0.03 (ns/0.0693)	**/0.0014	0.99 ± 0.12 (ns/>0.9999)	0.50 ± 0.06 (ns/0.9993)	ns/0.2020
5th	0.86 ± 0.05 (ns/>0.9999)	0.79 ± 0.03 (ns/>0.9999)	ns/>0.9999	2.11 ± 0.16 (ns/>0.9999)	1.49 ± 0.12 (ns/>0.9999)	***/0.0002	1.25 ± 0.19 (ns/0.9929)	0.70 ± 0.14 (ns/>0.9999)	ns/0.0855
6th	0.76 ± 0.03 (ns/>0.9999)	0.85 ± 0.02 (ns/>0.9999)	ns/>0.9999	1.91 ± 0.17 (ns/>0.9999)	1.55 ± 0.09 (ns/>0.9999)	ns/0.2369	1.15 ± 0.19 (ns/>0.9999)	0.70 ± 0.09 (ns/>0.9999)	ns/0.3150
7th	0.79 ± 0.06 (ns/>0.9999)	0.70 ± 0.03 (ns/0.9227)	ns/>0.9999	2.10 ± 0.15 (ns/>0.9999)	1.16 ± 0.05 (**/0.0013)	****/<0.0001	1.31 ± 0.17 (ns/0.9493)	0.46 ± 0.05 (ns/0.9957)	***/0.0003
8th	0.71 ± 0.05 (ns/0.9995)	0.70 ± 0.05 (ns/0.9227)	ns/>0.9999	2.16 ± 0.27 (ns/>0.9999)	1.28 ± 0.08 (*/0.0484)	****/<0.0001	1.45 ± 0.27 (ns/0.5547)	0.58 ± 0.12 (ns/>0.9999)	***/0.0002
9th	0.70 ± 0.05 (ns/0.9971)	0.55 ± 0.04 (*/0.0484)	ns/>0.9999	2.19 ± 0.25 (ns/>0.9999)	1.49 ± 0.08 (ns/>0.9999)	****/<0.0001	1.49 ± 0.24 (ns/0.4131)	0.94 ± 0.09 (ns/0.9996)	ns/0.0855
10th	0.61 ± 0.05 (ns/0.5384)	0.65 ± 0.06 (ns/0.5384)	ns/>0.9999	2.26 ± 0.14 (ns/>0.9999)	1.65 ± 0.15 (ns/>0.9999)	***/0.0003	1.65 ± 0.14 (ns/0.0562)	1.00 ± 0.17 (ns/0.9929)	*/0.0170
11th	0.63 ± 0.04 (ns/0.6516)	0.65 ± 0.03 (ns/0.5384)	ns/>0.9999	2.14 ± 0.19 (ns/>0.9999)	1.58 ± 0.12 (ns/>0.9999)	**/0.0014	1.51 ± 0.19 (ns/0.3271)	0.93 ± 0.12 (ns/0.9998)	*/0.0481
12th	0.75 ± 0.03 (ns/>0.9999)	0.75 ± 0.04 (ns/0.9995)	ns/>0.9999	1.90 ± 0.07 (ns/>0.9999)	1.88 ± 0.13 (ns/>0.9999)	ns/>0.9999	1.15 ± 0.09 (ns/>0.9999)	1.13 ± 0.17 (ns/0.8196)	ns/>0.9999
13th	0.85 ± 0.02 (ns/>0.9999)	0.75 ± 0.03 (ns/0.9995)	ns/>0.9999	2.06 ± 0.09 (ns/>0.9999)	1.89 ± 0.10 (ns/>0.9999)	ns/0.9995	1.21 ± 0.10 (ns/0.9986)	1.14 ± 0.11 (ns/0.7818)	ns/>0.9999
14th	0.85 ± 0.03 (ns/>0.9999)	0.75 ± 0.05 (ns/0.9995)	ns/>0.9999	1.65 ± 0.14 (ns/0.9664)	2.15 ± 0.14 (ns/0.9995)	**/0.0089	0.80 ± 0.14 (ns/>0.9999)	1.40 ± 0.14 (ns/0.0562)	*/0.0394
15th	0.76 ± 0.02 (ns/>0.9999)	0.81 ± 0.04 (ns/>0.9999)	ns/>0.9999	1.59 ± 0.10 (ns/0.5384)	2.28 ± 0.14 (ns/0.3341)	****/<0.0001	0.83 ± 0.09 (ns/>0.9999)	1.46 ± 0.17 (*/0.0200)	*/0.0211
16th	0.76 ± 0.03 (ns/>0.9999)	0.80 ± 0.04 (ns/>0.9999)	ns/>0.9999	1.63 ± 0.07 (ns/0.8532)	2.35 ± 0.14 (*/0.0484)	****/<0.0001	0.86 ± 0.06 (ns/>0.9999)	1.55 ± 0.16 (**/0.0039)	**/0.0087
17th	0.73 ± 0.03 (ns/>0.9999)	0.88 ± 0.04 (ns/>0.9999)	ns/>0.9999	1.54 ± 0.06 (ns/0.1876)	2.51 ± 0.08 (***/0.0002)	****/<0.0001	0.81 ± 0.07 (ns/>0.9999)	1.64 ± 0.11 (***/0.0006)	***/0.0006

R-βHB, R-beta-hydroxybutyrate; KEMCT, mix of ketone ester (KE) and medium-chain triglyceride (MCT) oil in a 1:1 ratio; ns, non-significant. *p < 0.05; **p < 0.01; ***p < 0.001; ****p < 0.0001.

### Age- and Sex-Dependent Alterations in Blood Glucose Levels

**FIGURE 3:**
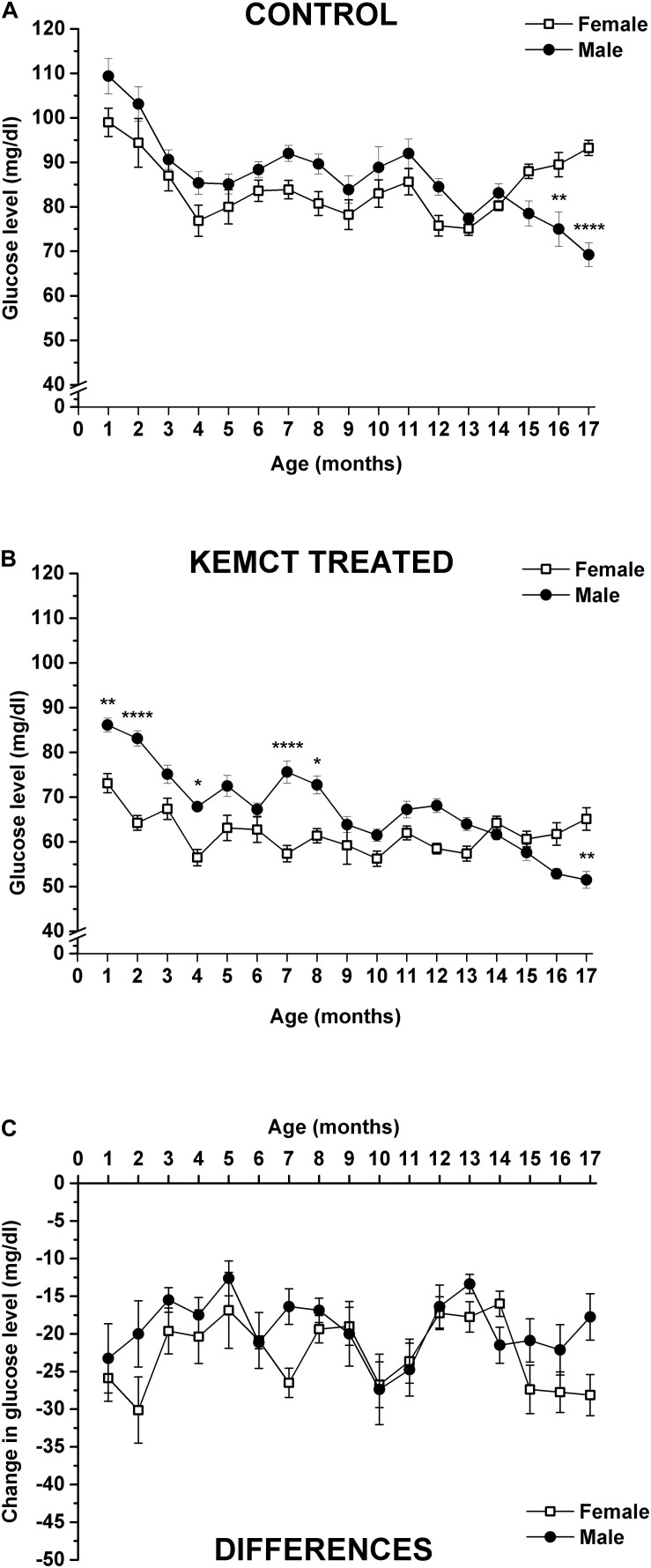
Sex-dependent changes in blood glucose levels. Control glucose levels were different (lower) in male rats, compared with female rats only at the 16th and 17th months **(A)**. KEMCT treatment-generated blood glucose levels were significantly lower in female than male rats at the 1st, 2nd, 4th, 7th, and 8th months, but it was higher at the 17th month **(B)**. Differences between KEMCT-evoked glucose levels and control glucose levels did not show significant alterations between sexes **(C)**. KEMCT, mix of ketone ester (KE) and medium-chain triglyceride (MCT) oil in a 1:1 ratio. *p < 0.05; **p < 0.01; ****p < 0.0001.

Blood glucose levels were significantly influenced by both age and sex (interaction of age and sex: *F*_33_,_476_ = 6.706, *p* < 0.0001; age: *F*_33_,_476_ = 49.30, *p* < 0.0001; sex: *F*_1_,_476_ = 46.28, *p* < 0.0001). Control blood glucose levels significantly decreased between the 4th and 14th months (except at the 11th month), compared with the baseline (1st month) in female rats, but between the 15th and 17th months, blood glucose levels were less decreased and reached baseline (1st month) level (Table 2, first column). A similar decrease in control blood glucose levels was observed in male rats, compared with the baseline, but this tendency was continued after the 14th month (between the 15th and 17th months) (Table 2, second column). Consequently, control glucose levels were significantly lower in male rats, compared with female rats at the 16th and 17th months (Figure 3A and Table 2, third column). After KEMCT gavage, the decrease in glucose level was similar to changes in control glucose levels in both sexes by age, compared with the baseline (Table 2, fourth and fifth columns). KEMCT treatment-generated glucose levels were lower between the 4th and 13th months in both female (significantly on the 4th, 7th, 9th, 10th, 12th, and 13th months) and male (between the 4th and 6th as well as between the 9th and 13th months) rats, compared with the baseline (1st month), but this tendency was continued only in male rats between the 14th and 17th months (Table 2, fourth and fifth columns). KEMCT treatment-generated blood glucose levels were significantly lower in female than male rats between the 1st and 8th months (except at the 3rd, 5th, and 6th months; Figure 3B and Table 2, sixth column), whereas a trend of higher glucose levels was demonstrated in female rats between the 14th and 17th months, reaching significance only at the 17th month (Figure 3B and Table 2, sixth column). Moreover, comparison of differences between KEMCT-evoked glucose levels and control glucose levels showed age- and sex-dependent differences (interaction of age and sex: *F*_16_,_238_ = 1.045, *p* = 0.4097; age: *F*_16_,_238_ = 2.985, *p* = 0.0001; sex: *F*_1_,_238_ = 9.836, *p* = 0.0019). However, differences between KEMCT-evoked glucose levels and control glucose levels did not show significant alterations either in sexes (Table 2, seventh and eighth columns) or between sexes (Figure 3C and Table 2, ninth column).

**TABLE 2:** Effect of age and sex on KEMCT-evoked changes in blood glucose levels.

**BLOOD GLUCOSE LEVELS (mg/dl; mean ± SEM)**									
**Age (month)**	**Control (2.5 g/kg water, gavage)**			**KEMCT treated (2.5 g/kg, gavage)**			**Differences (KEMCT treated – control)**		
	
	**Female (significance: compared to 1st month)**	**Male (significance: compared to 1st month)**	**Female vs. male: significance**	**Female (significance: compared to 1st month)**	**Male (significance: compared to 1st month)**	**Female vs. male: significance**	**Female (significance: compared to 1st month)**	**Male (significance: compared to 1st month)**	**Female vs. male: significance**
1st	99.00 ± 3.17 (−)	109.38 ± 4.01 (−)	ns/0.1081	73.13 ± 2.13 (−)	86.13 ± 1.58 (−)	**/0.0084	−25.88 ± 3.07 (−)	−23.25 ± 4.59 (−)	ns/>0.9999
2nd	94.38 ± 5.46 (ns/>0.9999)	103.13 ± 3.89 (ns/0.9982)	ns/0.3650	64.25 ± 1.63 (ns/0.8166)	83.13 ± 1.73 (ns/>0.9999)	****/<0.0001	−30.13 ± 4.39 (ns/>0.9999)	−20.00 ± 4.39 (ns/>0.9999)	ns/0.2900
3rd	87.00 ± 3.43 (ns/0.1790)	90.63 ± 2.19 (****/<0.0001)	ns/>0.9999	67.38 ± 2.39 (ns/0.9996)	75.13 ± 2.02 (ns/0.3490)	ns/0.6214	−19.63 ± 3.05 (ns/0.9906)	−15.50 ± 1.64 (ns/0.9313)	ns/0.9992
4th	76.88 ± 3.50 (****/<0.0001)	85.38 ± 2.53 (****/<0.0001)	ns/0.4245	56.50 ± 1.82 (**/0.0015)	67.88 ± 0.95 (***/0.0002)	*/0.0438	−20.38 ± 3.55 (ns/0.9977)	−17.50 ± 2.32 (ns/0.9962)	ns/>0.9999
5th	80.00 ± 3.84 (****/<0.0001)	85.13 ± 2.19 (****/<0.0001)	ns/0.9953	63.13 ± 2.83 (ns/0.5748)	72.50 ± 2.37 (*/0.0442)	ns/0.2389	−16.88 ± 5.04 (ns/0.8002)	−12.63 ± 2.31 (ns/0.5355)	ns/0.9988
6th	83.63 ± 2.41 (**/0.0068)	88.38 ± 1.75 (****/<0.0001)	ns/0.9987	62.75 ± 2.85 (ns/0.4867)	67.25 ± 1.45 (****/<0.0001)	ns/0.9995	−20.88 ± 3.71 (ns/0.9992)	−21.13 ± 0.83 (ns/>0.9999)	ns/>0.9999
7th	83.88 ± 2.07 (**/0.0091)	92.00 ± 1.79 (***/0.0006)	ns/0.5209	57.38 ± 1.84 (**/0.0044)	75.63 ± 2.48 (ns/0.4579)	****/<0.0001	−26.50 ± 1.94 (ns/>0.9999)	−16.38 ± 2.38 (ns/0.9759)	ns/0.2900
8th	80.75 ± 2.67 (***/0.0002)	89.63 ± 2.24 (****/<0.0001)	ns/0.3370	61.38 ± 1.58 (ns/0.2147)	72.75 ± 2.02 (ns/0.0561)	*/0.0438	−19.38 ± 1.81 (ns/0.9860)	−16.88 ± 1.62 (ns/0.9885)	ns/>0.9999
9th	78.25 ± 3.34 (****/<0.0001)	83.88 ± 3.09 (****/<0.0001)	ns/0.9815	59.25 ± 4.26 (*/0.0346)	63.88 ± 1.83 (****/<0.0001)	ns/0.9992	−19.00 ± 2.53 (ns/0.9759)	−20.00 ± 4.29 (ns/>0.9999)	ns/>0.9999
10th	83.00 ± 3.09 (**/0.0032)	88.87 ± 4.68 (****/<0.0001)	ns/0.9674	56.25 ± 1.69 (**/0.0011)	61.50 ± 1.35 (****/<0.0001)	ns/0.9932	−26.75 ± 3.03 (ns/>0.9999)	−27.38 ± 4.67 (ns/>0.9999)	ns/>0.9999
11th	85.63 ± 2.95 (ns/0.0561)	92.00 ± 3.23 (***/0.0006)	ns/0.9175	62.00 ± 1.54 (ns/0.3240)	67.25 ± 1.86 (****/<0.0001)	ns/0.9932	−23.63 ± 2.93 (ns/>0.9999)	−24.75 ± 3.50 (ns/>0.9999)	ns/>0.9999
12th	75.75 ± 2.29 (****/<0.0001)	84.50 ± 1.86 (****/<0.0001)	ns/0.3650	58.50 ± 1.19 (*/0.0158)	68.13 ± 1.55 (***/0.0002)	ns/0.1984	−17.25 ± 2.18 (ns/0.8482)	−16.38 ± 2.85 (ns/0.9759)	ns/>0.9999
13th	75.13 ± 1.54 (****/<0.0001)	77.38 ± 1.28 (****/<0.0001)	ns/>0.9999	57.38 ± 1.66 (**/0.0044)	64.00 ± 1.48 (****/<0.0001)	ns/0.8799	−17.75 ± 2.02 (ns/0.9007)	−13.38 ± 1.27 (ns/0.6651)	ns/0.9983
14th	80.25 ± 1.09 (****/<0.0001)	83.13 ± 2.02 (****/<0.0001)	ns/>0.9999	64.25 ± 1.51 (ns/0.8166)	61.63 ± 1.16 (****/<0.0001)	ns/>0.9999	−16.00 ± 1.67 (ns/0.6651)	−21.50 ± 2.39 (ns/>0.9999)	ns/0.9795
15th	88.00 ± 1.60 (ns/0.3490)	78.50 ± 2.82 (****/<0.0001)	ns/0.2179	60.63 ± 1.76 (ns/0.1210)	57.63 ± 1.77 (****/<0.0001)	ns/>0.9999	−27.38 ± 3.21 (ns/>0.9999)	−20.88 ± 2.87 (ns/>0.9999)	ns/0.9131
16th	89.50 ± 2.73 (ns/0.6903)	75.00 ± 3.86 (****/<0.0001)	**/0.0015	61.75 ± 2.51 (ns/0.2769)	52.88 ± 1.16 (****/<0.0001)	ns/0.3370	−27.75 ± 2.70 (ns/>0.9999)	−22.13 ± 3.34 (ns/>0.9999)	ns/0.9746
17th	93.25 ± 1.71 (ns/0.9996)	69.25 ± 2.64 (****/<0.0001)	****/<0.0001	65.13 ± 2.52 (ns/0.9349)	51.50 ± 1.89 (****/<0.0001)	**/0.0042	−28.13 ± 2.73 (ns/>0.9999)	−17.75 ± 3.07 (ns/0.9977)	ns/0.2544

KEMCT, mix of ketone ester (KE) and medium-chain triglyceride (MCT) oil in a 1:1 ratio; ns, non-significant. *p < 0.05; **p < 0.01; ***p < 0.001; ****p < 0.0001.

### Alteration of Body Weight by Age and Sex

**FIGURE 4:**
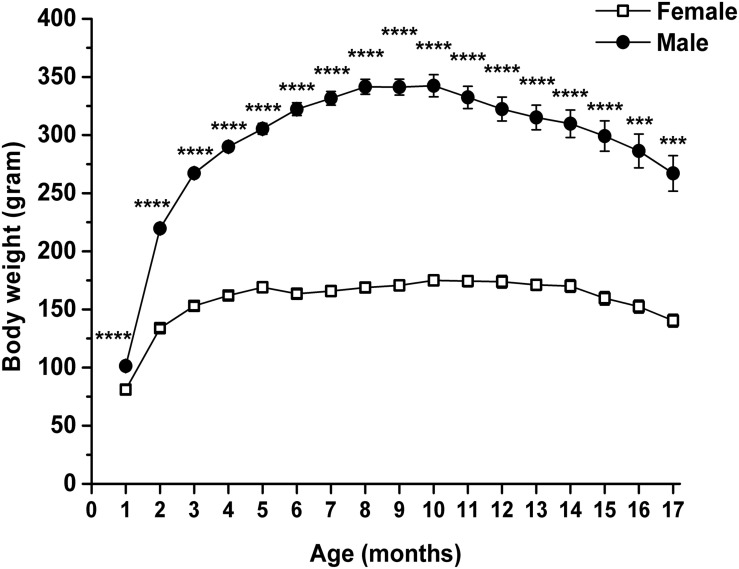
Body weight of female and male WAG/Rij rats. Body weight of male rats was significantly higher than the body weight of female rats. ***p < 0.001; ****p < 0.0001.

Body weight of rats was also significantly modulated by both age and sex (interaction of age and sex: *F*_16_,_224_ = 35.75, *p* < 0.0001; age: *F*_1_._762_,_24_._66_ = 173.1, *p* < 0.0001; sex: *F*_1_,_14_ = 294.8, *p* < 0.0001). Body weight of both female and male animals significantly changed between the 2nd and 17th months, compared with the baseline (1st month) (Table 3, first and second columns). Body weight increased until 10th month of age, and after that age, it gradually decreased. Moreover, the body weight of male rats was significantly higher than the body weight of female rats through the entire measuring period (Figure 4 and Table 3, third column).

**TABLE 3:** Effect of age and sex on body weight of WAG/Rij rats.

**Age (month)**	**BODY WEIGHT (gram; mean ± SEM)**		
	**Female (significance: compared to 1st month)**	**Male (significance: compared to 1st month)**	**Female vs. male: significance**
1st	81.00 ± 1.62 (−)	101.38 ± 1.43 (−)	****/<0.0001
2nd	133.88 ± 3.01 (****/<0.0001)	219.63 ± 2.52 (****/<0.0001)	****/<0.0001
3rd	153.00 ± 3.32 (****/<0.0001)	267.13 ± 3.02 (****/<0.0001)	****/<0.0001
4th	162.00 ± 3.18 (****/<0.0001)	289.88 ± 3.74 (****/<0.0001)	****/<0.0001
5th	169.00 ± 4.11 (****/<0.0001)	305.38 ± 4.65 (****/<0.0001)	****/<0.0001
6th	163.50 ± 3.51 (****/<0.0001)	322.25 ± 5.50 (****/<0.0001)	****/<0.0001
7th	165.75 ± 3.96 (****/<0.0001)	331.63 ± 5.89 (****/<0.0001)	****/<0.0001
8th	168.88 ± 3.80 (****/<0.0001)	341.50 ± 6.46 (****/<0.0001)	****/<0.0001
9th	170.63 ± 4.11 (****/<0.0001)	341.25 ± 6.81 (****/<0.0001)	****/<0.0001
10th	175.00 ± 4.46 (****/<0.0001)	342.38 ± 9.52 (****/<0.0001)	****/<0.0001
11th	174.38 ± 4.84 (****/<0.0001)	332.38 ± 9.56 (****/<0.0001)	****/<0.0001
12th	173.88 ± 5.39 (****/<0.0001)	322.38 ± 10.29 (****/<0.0001)	****/<0.0001
13th	171.13 ± 4.69 (****/<0.0001)	315.13 ± 10.56 (****/<0.0001)	****/<0.0001
14th	170.13 ± 5.41 (****/<0.0001)	309.75 ± 11.80 (****/<0.0001)	****/<0.0001
15th	159.63 ± 5.77 (****/<0.0001)	299.13 ± 13.08 (****/<0.0001)	****/<0.0001
16th	152.50 ± 5.58 (***/0.0001)	286.38 ± 14.51 (***/0.0001)	***/0.0002
17th	140.38 ± 5.51 (***/0.0003)	267.00 ± 15.25 (***/0.0004)	***/0.0005

***p < 0.001; ****p < 0.0001.

## Discussion

In this study, we demonstrated first by a long-term (17 months) study that the EKS KEMCT-generated changes in blood R-βHB and glucose levels can be modulated by age and sex.

Mix of KE and MCT can be metabolized in the gut and liver. KE hydrolyzes to AcAc and 1,3-butanediol by gut esterases, the component parts of which transport to the systemic bloodstream. AcAc can be transported from the intestinal lumen through intestinal epithelial cells by monocarboxylate transporters to the blood and may be utilized by cells of extrahepatic organs for energy production (Halestrap, 2013; Newman and Verdin, 2014). 1,3-Butanediol molecules further metabolize to ketone bodies, such as βHB, in the liver by alcohol dehydrogenases and aldehyde dehydrogenases. MCT oil can hydrolyze to medium-chain fatty acids in the gastrointestinal tract by lipases followed by direct absorption through the gut wall, and subsequently, medium-chain fatty acids may metabolize to ketone bodies in the hepatocytes (Tate et al., 1971; Clarke et al., 2012; Schönfeld and Wojtczak, 2016). Indeed, free fatty acids can convert into acyl-CoA in the liver, and acyl-CoA may metabolize to acetyl-CoA *via* mitochondrial β-oxidation. Acetyl-CoA may enter the Krebs cycle or convert into ketone bodies, such as βHB, by several metabolic enzymes such as acetoacetyl-CoA-thiolase, hydroxymethylglutaryl-CoA-synthase (Middleton, 1972; Hegardt, 1999), and βHB dehydrogenase (β-OHBD) (Lehninger et al., 1960; Kalapos, 2003). Ketone bodies can exit the liver *via* monocarboxylate transporters, enter the bloodstream and subsequently to various organs (e.g., brain, heart, and muscle), and serve for ATP synthesis (Halestrap, 2013; Newman and Verdin, 2014; Achanta and Rae, 2017).

It has been demonstrated that expression of monocarboxylate transporter 1 in the blood–brain barrier (Nehlig and Pereira de Vasconcelos, 1993; Gerhart et al., 1997) and cerebral glucose transporter 1 (GLUT1) and GLUT3 (Vannucci, 1994), as well as the activity of ketone body metabolic enzymes, such as β-OHBD, succinyl-CoA:3-ketoacid CoA transferase, and acetoacetyl-CoA thiolase (Page et al., 1971), may be age-dependent, not only in the rat brain but also in rat heart and kidney (Page et al., 1971). It has also been demonstrated that ketone body production of hepatocytes was higher in 8–10-week-old male rats, compared with female rats (Buc et al., 1986), and ketogenic capacity and β-OHBD activity were higher in male rats than in female rats under fasting condition (Teutsch, 1984), suggesting sex-dependent differences in ketone body metabolism of the liver. Testosterone was found to increase the expression of monocarboxylate transporter 1 and monocarboxylate transporter 4 in skeletal muscles, but not in the heart (Enoki et al., 2006). Female sex hormones can downregulate the hepatic expression of monocarboxylate transporter 1, whereas hepatic monocarboxylate transporter 4 expression may be modulated by testosterone (Cao et al., 2017). However, the levels and generation of ketone bodies increased more markedly in females than in males, not only in mice but also in humans under fasting conditions (Merimee et al., 1978; Jikumaru et al., 2007), likely by the modulatory effects of sex hormones (Morrow et al., 1981). More rapid and greater decrease in blood glucose level was also demonstrated in women, compared with men during starvation, whereas the basal level of glucose and βHB was similar in both sexes (Merimee et al., 1978), and higher hepatic glucose output was revealed in male rats during starvation (Gustavsson et al., 2010). Expression of hypothalamic GLUTs (e.g., GLUT1) increased by age in male, but not in female rats, and male adolescent rats had lower GLUT1 expression, than female adolescent rats (Kelly et al., 2014).

Previous studies show that WAG/Rij rats spontaneously develop absence epileptic seizures (spike-wave discharges: SWDs) after about 3 months of their age (Coenen and Van Luijtelaar, 2003). SWD number gradually increases by age, but significant sex differences in changes in SWD number were not demonstrated in WAG/Rij rats (Coenen and Van Luijtelaar, 1987, 2003). It was suggested that glucose metabolism may be higher in absence epileptic animals, such as GAERS rats (Genetic Absence Epilepsy Rats from Strasbourg), compared with non-epileptic control rats (Depaulis and Van Luijtelaar, 2006), but it may correlate with interictal periods, but not with the occurrence of SWDs (Nehlig et al., 1993). Moreover, glucose release from hepatocytes may increase by age in WAG/Rij rats (Teillet et al., 2001). However, we found that the control level of glucose (and its changes) was similar until approximately 14 months of their age in both sexes of WAG/Rij rats, but subsequently, glucose levels were gradually increased and decreased in female and male rats, respectively (Figure 3A). These results suggest that changes in blood glucose levels may be independent from changes in SWD number, and there may be sex-dependent differences in glucose utilization at least in older than 1-year-old WAG/Rij rats. Moreover, body weights of female and male WAG/Rij rats were significantly different (Figure 4 and Table 3), but similar control R-βHB and glucose (except between the 15th and 17th months) levels were demonstrated in both sexes (Figures 2A, 3A). These results suggest that likely there is no relationship between differences in body weight of female and male animals and alterations in not only the control but also KEMCT-evoked changes in blood R-βHB and glucose levels. However, the influence of body weight differences between sexes on KEMCT-generated effects cannot be ruled out entirely.

It has been demonstrated that ketosis and changes in the rates of ketone body utilization do not evoke changes in the relatively high activity and amount of ketone body metabolic enzymes in adults (Page et al., 1971; Williamson et al., 1971). Moreover, short periods of fasting or KD are not able to change blood–brain barrier permeability to ketone bodies likely because prolonged and high ketone body levels are needed for increased transport processes (Hawkins et al., 1971; Nehlig and Pereira de Vasconcelos, 1993; Morris, 2005). Based on these results, administration of EKSs (such as KEMCT) once a month for 17 months likely may not be able to change the activity of ketone body metabolic enzymes and blood–brain barrier permeability to ketone bodies. Thus, theoretically, changes in KEMCT-evoked increase in blood R-βHB levels by age and differences between female and male rats (Figures 2B,C and Table 1) may result likely from altered expression and activity of ketone body metabolizing enzymes and/or monocarboxylate transporters in different organs between the 1st and 17th months (but not from direct and acute effects of KEMCT on ketone body metabolism or transport). Theoretically, similar to ketone bodies, changes by age and sex in glucose utilization month by month (but not an acute effect) may result in KEMCT-generated decrease in blood glucose concentrations (Figure 3B and Table 2). However, more studies are needed to find the exact links between the KEMCT-evoked changes in blood R-βHB and glucose levels and changes in the activity of metabolic enzymes and transporters of different organs during aging (as putative background of KEMCT-generated effects) in animals with different ages and in both sexes.

The influence of age- and sex-generated effects on glucose and ketone body metabolic enzymes and transporters under normal physiological and pathophysiological conditions, as well as after EKS administration through a wide age range of rats (month by month), has not been well studied. Moreover, actual βHB and glucose levels and utilization may reflect alterations not only in the liver but also in extrahepatic tissues by age (Page et al., 1971; Edmond, 1974), and the mechanism of digestion and absorption of fat (and, theoretically, EKSs) can be changed in the gut by age (Henning and Kretchmer, 1973). Thus, interpretation of our results and the explanation of the complex background processes leading to modulation of KEMCT-evoked changes in blood R-βHB and glucose concentrations by age and sex are difficult. However, our results strengthened the conclusions of previous studies above (Hawkins et al., 1971; Page et al., 1971; Merimee et al., 1978; Gerhart et al., 1997; Buc et al., 1986), suggesting that (i) ketone body and glucose production and utilization is maintained during the lifetime in rats, which may be modulated by age and sex; (ii) the level of both ketone bodies (R-βHB) and glucose may decrease by age after weaning; and (iii) basal levels of R-βHB and glucose are similar in females and males (Figure 2A, 3A and Tables 1, 2). In addition, our recent study markedly extends the previous results on EKS administration-generated effects on blood R-βHB and glucose levels pointing out that (i) KEMCT-evoked increase in blood R-βHB levels was significant in both sexes over 17 months, compared with the control (Figures 1A,B); (ii) the rate of increase was sex-dependent (Figures 2B,C and Table 1); iii) KEMCT-generated effects on blood R-βHB levels changed by age only in male rats, compared with the baseline (1st month) (Table 1); (iv) KEMCT administration not only significantly increased blood R-βHB levels but also significantly decreased the blood glucose levels in both sexes between the 1st and 17th months, compared with the control (Figures 1C,D), suggesting ketosis-induced hypoglycemia; (v) the rate of decrease in blood glucose levels was also sex-dependent (Figure 3B and Table 2); and (vi) KEMCT-generated influences on blood glucose levels changed by age in both sexes, compared with the baseline (1st month) (Table 2). Based on these results, we speculated that various influences could be generated on physiological and pathophysiological processes by the same doses of EKSs *via* distinct alteration of ketone body and/or glucose levels in males and females at different ages. However, blood levels of ketone bodies may not accurately reflect their levels in the brain (or in other organs) and their effect (efficacy) on physiological and pathophysiological processes, because ketone body transport could be saturated (Pellerin et al., 1998); thus, alleviating effects of ketone bodies (ketosis) on different diseases may not be necessarily increased by a higher level of ketosis, for example, in females compared with males (Figure 2B) and in adult males compared with younger ones (Table 1). In addition, a very low level of EKS-evoked ketosis may be ineffective and an exaggerated level of ketone bodies may not only be inefficacious but also harmful. Indeed, our previous study showed that, for example, to evoke an alleviating effect on impaired motor functions, generation of a certain range of blood ketone body levels is needed (Ari et al., 2020). However, these results suggest that different doses of EKSs may be needed for safe and effective treatment of different age groups and in both genders.

## Conclusion

In conclusion, emerging science on metabolic therapies, such as EKSs, suggests the potential of these compounds to enhance quality of life and for the treatment of different neurometabolic and age-related diseases. The present study suggests that the EKS-evoked effects on blood R-βHB and glucose levels may be modulated by both age and sex. Thus, the age- and gender-dependent modulatory effects of EKSs on blood R-βHB (ketosis) and glucose concentrations should be taken into consideration during the administration of metabolic-based therapies for the treatment of different diseases. Moreover, monitoring of blood ketone body and glucose levels of EKS users may be important in order to find safe and effective doses of EKSs for different age groups and in both genders. However, further studies are needed to reveal the exact mechanism(s) of action of ketone body and glucose production (metabolism) and utilization during aging and the potential gender-evoked modulatory processes on EKS-induced blood βHB and glucose levels not only in animals but also in human subjects.

## Data Availability Statement

The raw data supporting the conclusions of this article will be made available by the authors, without undue reservation.

## Ethics Statement

The animal study was reviewed and approved by the Hungarian Act of Animal Care and Experimentation.

## Author Contributions

ZK designed the experiments, acquisition, analysis, and interpretation of the data, and wrote the manuscript. BB contributed to the acquisition of the data. DD’A revised the manuscript. CA analyzed the data and wrote the manuscript. All authors read and approved the final manuscript.

## Conflict of Interest

International Patent # PCT/US2014/031237, University of South Florida for DD’A: “Compositions and Methods for Producing Elevated and Sustained Ketosis”. DD’A and CA are co-owners of the company Ketone Technologies LLC, a company specialized on scientific research, consulting, and public speaking. All authors declare that there are no additional conflicts of interest.

## Abbreviations

AcAc: acetoacetate
β-OHBD: β HB dehydrogenase
CNS: central nervous system
EKSs: exogenous ketone supplements
GLUT: glucose transporter
KDs: ketogenic diets
KE: ketone ester
KEMCT: mix of KE and MCT oil
MCT oil: medium-chain triglyceride oil
R- β HB: R-beta -hydroxybutyrate
SWDs: spike-wave discharges
WAG/Rij: Wistar Albino Glaxo/Rijswijk.
